# Nanotechnology-Based Strategies for Effective and Rapid Detection of SARS-CoV-2

**DOI:** 10.3390/ma14247851

**Published:** 2021-12-18

**Authors:** Koena L. Moabelo, Darius R. Martin, Adewale O. Fadaka, Nicole R. S. Sibuyi, Mervin Meyer, Abram M. Madiehe

**Affiliations:** Department of Science and Innovation (DSI)/Mintek Nanotechnology Innovation Centre (NIC), Biolabels Research Node, Department of Biotechnology, University of the Western Cape (UWC), Bellville 7535, South Africa; 3780365@myuwc.ac.za (K.L.M.); 3517594@myuwc.ac.za (D.R.M.); afadaka@uwc.ac.za (A.O.F.); nsibuyi@uwc.ac.za (N.R.S.S.)

**Keywords:** aptamer, COVID-19, diagnostics, nanoparticles, point-of-care testing, SARS-CoV-2

## Abstract

The coronavirus disease 2019 (COVID-19) pandemic has gained worldwide attention and has prompted the development of innovative diagnostics, therapeutics, and vaccines to mitigate the pandemic. Diagnostic methods based on reverse transcriptase-polymerase chain reaction (RT-PCR) technology are the gold standard in the fight against COVID-19. However, this test might not be easily accessible in low-resource settings for the early detection and diagnosis of severe acute respiratory syndrome coronavirus 2 (SARS-CoV-2). The lack of access to well-equipped clinical laboratories, requirement for the high level of technical competence, and the cost of the RT-PCR test are the major limitations. Moreover, RT-PCR is unsuitable for application at the point-of-care testing (PoCT) as it is time-consuming and lab-based. Due to emerging mutations of the virus and the burden it has placed on the health care systems, there is a growing urgency to develop sensitive, selective, and rapid diagnostic devices for COVID-19. Nanotechnology has emerged as a versatile technology in the production of reliable diagnostic tools for various diseases and offers new opportunities for the development of COVID-19 diagnostic systems. This review summarizes some of the nano-enabled diagnostic systems that were explored for the detection of SARS-CoV-2. It highlights how the unique physicochemical properties of nanoparticles were exploited in the development of novel colorimetric assays and biosensors for COVID-19 at the PoCT. The potential to improve the efficiency of the current assays, as well as the challenges associated with the development of these innovative diagnostic tools, are also discussed.

## 1. Introduction

For the first time in decades, the entire world suffered a huge blow served by COVID-19, which brought all systems to a standstill for almost 4 months. The only sector that was fully functional was the health sector in an attempt to flatten the curve and cater to those who urgently needed health care. The countries hardest hit by the pandemic had their health systems stretched to capacity in the first three waves and have been struggling to get the operational systems back to normal. By now, it is clear that COVID-19 is certainly not going anywhere anytime soon as the fourth wave is currently underway. Unfortunately, for survivors, COVID-19 repercussions are long lasting, and immunity against the disease is not guaranteed as re-infections have been reported. With inadequate protection from the immune system or a lack of effective treatments, the virus will continue to thrive due to mutations in the spike (S) protein of the SARS-CoV-2 [1]. By September 2020, new SARS-CoV-2 variants were identified, where this particular variant was related to mink farming and first reported in Denmark. The strain was detected in the United Kingdom and South Africa in December 2020, and, as of 24 January 2021, it had spread to more than 60 countries globally [1]. Since then, other deadly and highly infectious SARS-CoV-2 variants were reported in Brazil, South Africa, and India. At the time, these countries were red-flagged and their citizens were banned from travelling to several countries in Europe and the USA. So far, the alpha, beta, gamma, delta, and omicron variants have been identified as SARS-CoV-2 variants of concern (VOC). As a result of the genetic mutations, these variants have increased transmission rates, virulence, and are more resistant towards current treatments and vaccines [2,3]. The omicron variant, which was recently reported in South Africa, the UK, France, Israel, Belgium, the Netherlands, Germany, Italy, Australia, Canada, and Hong Kong, has more mutations and might be more problematic than the other VOC.

To combat this disease, effective and reliable diagnostic systems are needed. Systems that can identify individuals that have contracted the virus at the onset are needed so that they can be isolated to prevent the rapid spread of the virus. Currently, the disease can only be detected after 7–14 days of infection and only for those who present symptoms. The symptoms are not disease specific since other respiratory diseases can be mistaken for COVID-19. Patients, therefore, need to wait until the virus is fully manifested within their bodies. The RT-PCR method is regarded as the gold standard for testing COVID-19 infections. However, these tests are expensive, not always specific, and take 3–4 h to produce results. Rapid diagnostic assays that make use of antibodies have also been reported. The antibodies are, however, not stable, not specific, and can provide false-positive results [4,5].

As such, an effective diagnostic tool that is rapid, sensitive, specific, cost-effective, and easy to use at the point of care (PoC), either at home or in resource-limited settings, is crucial for the management of the disease and the support of quarantine programs. This review highlights the epidemiology of COVID-19, the currently used tests for COVID-19 diagnosis and their limitations, and discusses potential targets arising for SARS-CoV-2 diagnostics. With the recent advances in nanotechnology having provided a depth of insight and new opportunities for the application of nanomaterials in biological analysis and disease diagnosis, the review further discusses the recently developed nano-based diagnostics, their principles, and future perspectives as companion tools for a future paradigm at the PoC.

## 2. COVID-19 Epidemiology

In December 2019, the initial familial cluster of patients presenting clinical signs and symptoms of pneumonia were reported in Wuhan, China, and the causative agent was later identified as a novel SARS-CoV-2 [6,7]. On 30 January 2020, the World Health Organization (WHO) declared the COVID-19 outbreak a Public Health Emergency of International Concern [8,9]. The COVID-19 pandemic had spread to ~223 countries across the seven continents, and, as of 29 November 2021, SARS-CoV-2 infections have been reported in over 261 million individuals, with recoveries and mortality of 236 million and approximately 5.3 million, respectively [10].

Full-genome sequencing and phylogenetic analysis indicated that SARS-CoV-2 belongs to the same sub-genus beta coronavirus as other bat-derived CoVs, such as SARS and the Middle East respiratory syndrome (MERS) CoVs, but arose from different clades [11]. The CoVs are enveloped, positive single-stranded RNA viruses [12] that belong to the order Nidovirales, family Coronaviridae, and subfamily Coronavirinae. They are divided into four genera: the alpha, beta, gamma, and delta. They can cause diseases in humans and animals, varying from mild to severe. The alpha and beta CoVs cause infections in mammals, while the gamma and delta (but not exclusively) are associated with infections in birds [13]. The human alpha (HCoV-229E 229E and HCoV-NL63) and beta (HCoV-OC43 and HCoV-HKU1) CoVs have been reported to cause influenza-like illness or pneumonia in humans [14,15,16]. Two zoonotic beta CoVs have also been reported to cause severe disease in humans, including the SARS-CoV and MERS-CoV [14,15,16,17,18].

Despite the similarities with the other CoVs, the pathogenesis of SARS-CoV-2 is still unclear [19], making its infections more challenging to treat and control. One theory is that SARS-CoV-2 originated and evolved from animals. At the moment, prevention is through the use of personal protective equipment, practice of good hygiene, and keeping a social distance of 1.5 to 2 metres, and, ultimately, through the use of recommended vaccines [6]. More information about the SARS-CoV-2 epidemiology, pathophysiology, diagnosis, and management was reviewed by Fadaka et al. [6].

### 2.1. SARS-CoV-2 Clinically Validated Protein Targets for Diagnosis

**Figure 1:**
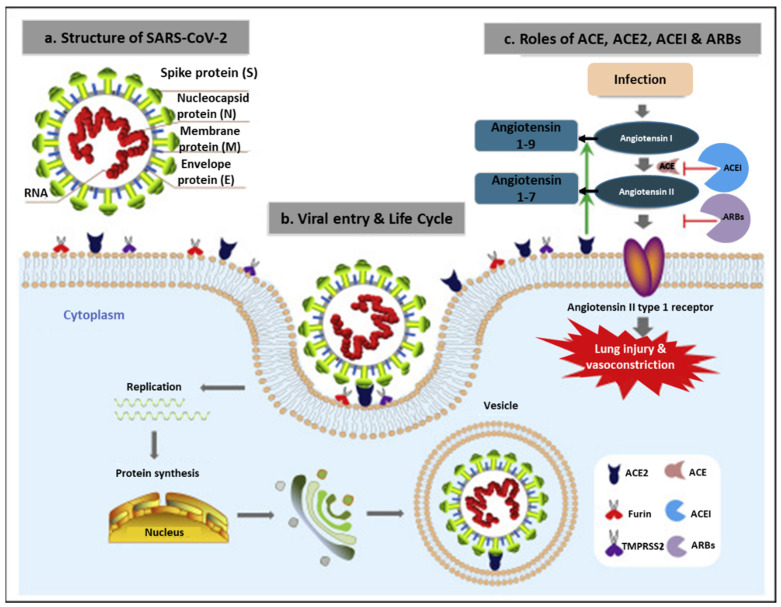
Structural component of the novel SARS-CoV-2 and mode of host penetration. (**a**) SARS-CoV-2 is composed of the viral RNA and structural proteins (S, E, M, N) that are shared amongst the CoV family; (**b**) viral entry and life cycle into the host; and (**c**) the roles of angiotensin-converting enzyme (ACE), ACE-2, ACE inhibitors (ACEIs), and angiotensin receptor blockers [21].

Diagnosis of SARS-CoV-2 infections is targeted at its structural components shown in Figure 1a. All the CoVs (SARS-CoV, MERS-CoV, and SARS-CoV-2) have similar structural features. They are spherical in shape with a diameter of ~100 nm, and are made up of four structural proteins: S, membrane (M), envelope (E), and the nucleocapsid (N) proteins. The N proteins form part of the inner viral genomic RNA, while the other structural proteins (S, E, and M) are embedded on the lipid bilayer and constitute the viral envelope [6,12,13,20,21]. The N, E, S, RNA-dependent RNA polymerase (RdRp), and the open reading frame (ORF) 1ab genes are used as gene targets for the detection of the SARS-CoV-2 infections by RT-PCR, while the S and N proteins are the main antigenic targets for antibody-based detection systems [8,20].

#### 2.1.1. Spike (S) Glycoprotein

The S Glycoprotein is an essential viral protein for the initiation of CoV infections through the receptor-binding and fusogenic regions, as shown in Figure 1b [13]. The receptor-binding domain (RBD) allows for the attachment of the virus to the host cells via the angiotensin-converting enzyme (ACE)-2 receptor [12]. The fusogenic region, on the other hand, enables viral entry and fusion into the host cells [22]. Viral entry is thus dependent on successful viral attachment to the ACE-2 receptor through the RBD. The detailed mechanism is described by Shang et al. [23]. Compared to the other CoVs, SARS-CoV-2 uses the S protein to bind more strongly to the host cells. This explains why SARS-CoV-2 spreads more easily from person to person than other viruses within this family. This protein is used clinically as one of the biomarkers for detection of COVID-19 infections. Antibodies that bind to the S protein have been reported to block viral entry into the host cell. However, over time, mutations in this protein can interfere with antibody binding, enabling the virus to escape the immune system [24].

#### 2.1.2. Membrane (M) Protein

These are the most abundant structural proteins that provide shape and give the membrane its 3D structure [25,26]. The M protein interacts with the N protein to enclose the RNA genome [27,28]. It contains three transmembrane domains, flanked by a short amino terminus outside the virion and a long carboxy terminus inside the virion [29]. The M proteins vary within the CoV family due to their highly diverse amino acid contents. Compared to the M protein of SARS-CoV, the one in SARS-CoV-2 is not amenable to mutations [26]. SARS-CoVs have been reported to induce apoptosis in virus-infected cells through this protein [30]. The virus-infected cells show distinctive apoptotic features, which include cell shrinkage, chromatin condensation, and formation of apoptotic bodies [31]. This feature makes this protein a target for therapeutic intervention in addition to its clinical use as a diagnostic biomarker in various immunological assays.

#### 2.1.3. Envelop (E) Protein

The E proteins are the smallest of the structural proteins and are important in regulating the viral replication in the host cells, including its entry, assembly, and release [32]. The E protein is made up of three important regions: a short hydrophilic amino terminal, a large hydrophobic membrane region, and a C-terminal region [33]. These three regions provide stability to the virus–host cell interactions and serve as an ion channel that allows movement of viral ions into the host cells [34]. In fact, inactivation of this protein has been reported to alter the virus morphology and ultimately change the virulence of CoVs [35]. Molecular tests targeting this protein were made available on 13 January 2020 [20,36]; however, these tests were reported to contain contaminations arising from poor lab practices that yielded false positive results in asymptomatic patients [37]. Targeting the E protein using other techniques can improve the sensitivity and specificity of the tests for the diagnosis of CoVs.

#### 2.1.4. Nucleocapsid (N) Protein

The N protein is a multifunctional protein with three distinct and conserved regions: the N-terminal RNA-binding domain (NTD), the C-terminal RNA-binding domain (CTD), and the serine/arginine rich region (SR), also known as the linker region [38]. These regions play important roles in binding to the viral genome, promote the interaction of the M protein with the host cell during viral assembly, and are involved in the regulation of the viral transcription [39,40]. The NTD, which facilitates attachment of the 3′ viral genome, differs in length and sequences amongst the CoVs [41]. The CTD, although its roles are not fully explored, was reported to contain a mutation in SARS-CoV-2 at position 334. Other mutations were also reported in the N protein of SARS-CoV-2, including two in the intrinsically dispersed region (positions 25 and 26) and one in the NTD (position 103) and SR (position 217) regions [26]. The SR regions play crucial roles in cell signalling [42,43,44] and act as an antagonist to prevent recognition by the host immune cells, such as interferon (IFN) and RNA interference [45]. Any of the N protein regions could be ideal targets for the diagnosis of the SARS-CoV-2.

#### 2.1.5. RNA-Dependent RNA Polymerase (RdRp)

RdRp, also known as Nsp12, is the main polymerase responsible for SARS-CoV-2 RNA polymerisation, and it is essential for viral replication [20,46]. It has approximately 932 amino acid residues and is made up of two conserved domains, the nidovirus RdRp-associated nucleotidyltransferase (NiRAN) and the polymerase domains [47]. The NiRAN domain is highly conserved in all nidoviruses for nucleotidylation activities [47]. The RdRp of SARS-CoV and that of SARS-CoV-2 are reported to be 94% identical [48] and were also used as targets for the diagnosis and management of the spread of the CoV infections.

#### 2.1.6. Non-Structural Proteins (nsps) and Accessory Proteins

Other proteins that have also been reported to play roles in SARS-CoV-2 transcription include 15 nsps and 8 accessory proteins [49]. Unlike the accessory proteins of other CoVs, SARS-CoVs have shorter ORF3b and longer ORF8b proteins. The 15 nsps of SARS-CoV-2 include nsp1–10 and nsp12–16, while the eight accessory proteins are ORF14, 3a, 3b, p6, 7a, 7b, 8b, and 9b [26]. While mutations have been reported in other CoVs, none have been reported in the nsp7, nsp13, envelope matrix, p6, and 8b of the SARS-CoV-2 [26]. These proteins could be suitable targets for diagnostics to curb the spread of SARS-CoV-2.

### 2.2. Diagnosis of SARS-CoV-2 Infections

The types of tests made available for the clinical diagnosis of SARS-CoV-2, after the WHO declared COVID-19 a pandemic, are listed in Table 1. The tests are used to detect the presence of the viral RNA, antigens, or antibodies through nucleic acid amplification tests (NAATs: RT-PCR, next generation sequencing) and immunological assays [12,50,51]. The RT-PCR method is the gold standard for detection of SARS-CoV-2 in SARS-CoV-2-infected RNA samples obtained from the respiratory tract specimens [20,46,52]. The assay is often used in combination with other methods (immunological methods and chest X-ray imaging) at different stages of the disease for an improved diagnostic efficiency of SARS-CoV-2 [53,54]. For example, the RT-PCR and IgM antibody tests can be used for the diagnosis of SARS-CoV-2 immediately from the 1st up to the 39th day after the onset of the symptoms [55], while the chest computed tomography (CT) scan and ultrasonography can be used in the pneumonia stage of COVID-19 infection [56,57,58]. Although the chest CT scans have higher sensitivity (97.2%) than RT-PCR (84.6%) [57], the CT findings can be non-specific and overlap with other viral infections, such as influenza and H1N1 [59].

#### 2.2.1. Nucleic Acid Amplification Tests (NAATs)

The RT-PCR is one of the routine NAATs used for COVID-19, as recommended by the WHO [9,60]. The test is used to detect the sequence of SARS-CoV-2 genes at various sites of the target genes (N, S, E, ORF1ab, RdRP, and nsp14). The N gene is a common target for diagnosis of COVID-19 in most countries, detecting up to three regions of this gene; one region is common for all CoVs and two regions are specific to SARS-CoV-2. The N gene can be tested separately or alongside other gene targets [9,12].

The RT-PCR has been successfully used in detecting the presence of SARS-CoV-2 [20,46,52]; however, it might not be convenient for the diagnosis of highly infectious diseases (such as COVID-19). The turnaround time for the test is quite long (2–6 h) [9,51] and requires the use of highly specialized instrumentation [61] and biosafety conditions. False negative results have been reported for RT-PCR [51]. Various NAAT-based systems with a potential for diagnostics of COVID-19 at the PoC are being developed. A batch of fluorescence-based quantitative kits and sequencing systems have recently been approved by the China Food and Drug Administration [9,12]. These include nested RT-PCR, droplet digital PCR, and loop-mediated isothermal (LAMP) amplification. Their sensitivity is 10-fold higher than RT-PCR, they are compatible with any type of sample (lower or upper respiratory tract samples), and they are still effective at very low viral loads. Systems such as LAMP are species-specific and can amplify the viral RNA using one temperature, resulting in a colorimetric product that can be visually analysed [9,12,60].

#### 2.2.2. Chest CT and Ultrasonography Imaging

The chest radiography, CT, and ultrasonography are used at the pneumonia stage to identify the graphical features of SARS-CoV-2 in COVID-19 patients [51,56,57,58]. During this stage, the CT test has higher sensitivity (97.2%) than the NAATs (84.6%) [51,57]. The test can be done within 15 to 120 s [9]. The use of artificial intelligence with the CT tests in clinics has reduced the detection time to 20 s, improved the accuracy rate of the analysis [62], and, ultimately, improved the diagnosis efficiency of the imaging techniques. However, interference has been reported for the CT imaging of SARS-CoV-2 as its features overlap with those of influenza and H1N1 [59,60]. However, these techniques are not only expensive [61] but are yet to be approved for clinical use [55]. Moreover, this technique may not be readily available for screening at the PoC and in low-resource settings.

#### 2.2.3. Immunological Tests

Immunological assays, such as the ELISA, CLIA, FIA, LFIA, and neutralizing antibody assays, are not standard tests for the detection of SARS-CoV-2 but are useful confirmatory tests in circumstances where the RT-PCR tests of symptomatic patients are negative [12,60]. This is because COVID-19 infected patients have been reported to have acute immune responses [22]. The antibodies (IgA, IgM, and IgG) are produced in the mucosal and blood samples of COVID-19 patients at varying infection times. The IgA production in the mucus secretions is detected within 6 to 8 days of infection. IgM appears in the blood within 5 to 7 days of infection and serves as an indicator for new infections. IgG is detectable from 10 to 15 days of infection and lasts for months after recovery [12,51]. Immunological tests have been reported for the detection of SARS-CoV-2 antigens, viz., the N, S, and ORF1ab proteins [9]. Despite the potential use of the antibody tests from the 1st to the 39th day after the onset of the COVID-19 symptoms, these tests can generate false negative results since the dynamics of antigen production and secretion have not been well established [55].

## 3. Advances towards Development of Rapid Diagnostics for SARS-CoV-2

Owing to the large scale and rapid increase of this unprecedented outbreak, preventative measures through quick and reliable diagnostics are vital to improve patients’ treatment outcomes and to manage the spread of the disease [63]. The early and rapid detection of COVID-19 could allow patients to get treatment sooner, minimize transmission, and prevent laboratory testing backlogs [64]. The emergence of asymptomatic carriers and mutant strains of SARS-CoV-2 have brought great challenges to control COVID-19, further aggravating the spread of the disease [48]. In addition, proper diagnosis and early detection in low-resource settings has proven to be a challenge due to a lack of access to well-equipped clinical laboratories and the requirement for a high level of technical competence [65]. Therefore, there is an urgent need to develop reliable, highly sensitive, and selective diagnostic systems for SARS-CoV-2 to better control the spread of the disease.

Globally, companies are competing to dominate the market by developing PCR- and ELISA-based rapid commercial detection kits [50]. With the emergence of new strains, which are deadlier than the original strain, efforts to improve the performance of the existing diagnostic systems have been realized by bringing in new technologies to these systems. The clustered regularly interspaced short palindromic repeats (CRISPR) and nanotechnology-based diagnostics come highly recommended and have shown the possibility to develop novel strategies for rapid detection of SARS-CoV-2 [66]. The Cas13-based specific high-sensitive enzymatic reporter unlocking (SHERLOCK) platform combines the capability of NAAT-based isothermal recombinase polymerase amplification at 22 to 45 °C and sequence-specific detection probes. Cas13-based SHERLOCK was used in the detection of problematic viruses, such as Zika and Dengue viruses [67], and, more recently, SARS-CoV-2 [68]. The Cas13-based assay was used in a blind study to detect the S gene in the clinical nasopharyngeal RNA samples that were confirmed COVID-19 positive by the RT–PCR assay that targeted the N gene [68]. This platform showed 100% specificity and sensitivity, and 97% for the fluorescence and lateral flow-based Cas13-based assays, respectively. The system was highly specific for the target genes, viz., S, N, and Orf1, with no cross-reactivity towards other human CoVs [68].

Nanotechnology has also raised the bar in developing rapid, robust, sensitive, and cost-effective diagnostic systems [46,69]. Nanomaterials can be integrated in various parts of existing sensing platforms in order to offer innovative diagnostic assays [70]. To date, a number of nanotechnology-based diagnostic systems have been developed to detect diseases, toxins, pathogens, and viruses [71,72,73,74]. It is believed that these emerging nanotechnologies could be adopted in diverse settings throughout the globe, from managing the current (COVID-19, Ebola) and future health threats while securing both the human and economic wellness.

### 3.1. Nanomaterial-Based Diagnostic Systems for COVID-19

Nanotechnology is a multidisciplinary field that includes the design, production, and application of materials at a nanometre scale. This field has revolutionized and advanced various intersecting scientific fields through provision of new opportunities for the application of nanomaterials in various systems, including biological analysis and disease diagnosis [75,76]. Nanomaterials have gained worldwide attention in diagnostics and therapeutics due to their unique physicochemical properties that are different from those observed in bulk materials [77]. Their small size and large surface area enhance their surface reactivity, quantum confinement effects, electrical conductivity, and magnetic properties, which have made them potential tools in developing innovative diagnostic systems [78,79,80]. Moreover, they can be easily functionalized with other biomolecules. To increase their specificity, different biorecognition molecules, such as antibodies, peptides, aptamers, nanobodies, etc., can be conjugated to their surface [81,82]. Due to these properties, various nanomaterials, such as gold nanoparticles (AuNPs), magnetic NPs, upconverting NPs, and quantum dots (QDs), have been engineered to detect molecular targets of interest for diagnostic purposes. Compared to conventional probes that suffer from instability and photobleaching, NPs possess unique properties that can counter these effects. Thus, taking advantage of the above-mentioned properties, nano-enabled optical, magnetic, fluorescence, and optomagnetic biosensors have been developed for the detection of various viruses, including SARS-CoV-2. Table 2 summarizes the various nano-based methods used for the diagnosis of SARS-CoV-2.

#### 3.1.1. Colorimetric Assays

Colorimetric assays have been extensively used for rapid detection of various diseases due to their simplicity, quick response, high sensitivity, and mostly visually detected responses [93,94,95]. Binding of the target biomarkers to the analyte molecule triggers an enzymatic or chemical reaction that will result in a signal, which is indicated by a colour change. These changes are detected visually in case of LFAs, and the signals are quantifiable by measuring the absorbance at a specific wavelength [96].

Metallic nanoparticles (MNPs) exhibit strong localized surface plasmon resonance (LSPR) properties that have been widely leveraged for the fabrication of colorimetric sensors [97]. Among the available MNPs, AuNPs have been widely explored in colorimetric assays. AuNPs produce a colourful signal that can be visualized with the naked eye without the use of advanced instruments. Generally, a colloidal AuNP solution has a ruby-red colour due to the LSPR phenomenon that is highly dependent on the interparticle distance [83,85,98]. The binding of the target biomarker molecules to the AuNPs functionalized with biorecognition elements induces a distinct shift in their LSPR, resulting in the change of colour from ruby-red to blue [98,99]. The colour can be visually detected or measured with an optical device. In an ELISA or LFA tests, the intensity of the colour is correlated to the amount of sample adsorbed to AuNP-conjugates [100,101].

##### AuNP-Based Colorimetric Assay

**Figure 2:**
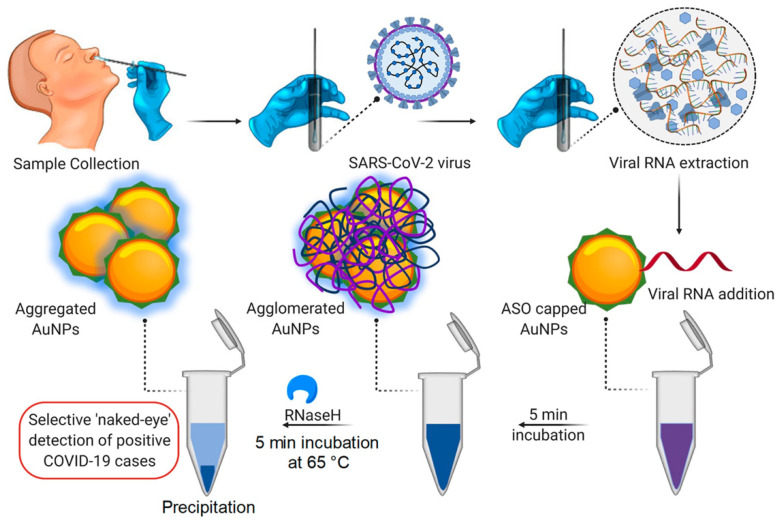
AuNP-based colorimetric assay for SARS-CoV-2 in RNA samples from COVID-19 patients. ASOs capped AuNPs were incubated with the RNA samples for 5 min; blue colour was visually observed in the positive samples [83].

AuNP-based colorimetric assays capable of detecting the SARS-CoV-2 RNA targets [83,84] and IgM [85] have been reported. These assays offer high sensitivity and specificity, with the AuNPs producing a coloured signal that is visible to the naked eye in a test tube [83,84] or LFA [85]. Their performance was comparable to that of clinical standard tests, with reduced turnaround time of 10 to 15 min (Figure 2). In the presence of the target RNA sequence of SARS-CoV-2, the antisense oligonucleotides (ASOs) bound to the N gene of SARS-CoV-2 leading to AuNP aggregation and a colour change. Upon the addition of RNaseH, the RNA strand was cleaved from the RNA–DNA hybrid, which led to the formation of a visually detectable precipitate as a result of AuNP agglomeration. The AuNP-based assay was also used to assess the presence of N gene in the viral RNA samples extracted from Vero cells infected with SARS-CoV-2 obtained from an oropharyngeal swab of a COVID-19 patient. Incubation of the ASOs-capped AuNPs with the SARS-CoV-2 RNA samples induced a blue colour change that was visually detected within 10 min. The blue colour served as a confirmatory test for the presence of SARS-CoV-2. The test was sensitive with a limit of detection (LOD) of 0.18 ng/μL for the SARS-CoV-2 RNA. The biosensor demonstrated high specificity for the target by not showing any colour change after incubation with MERS-CoV RNA [83].

This assay was further tested on human nasopharyngeal RNA samples to detect the RdRp gene sequence of SARS-CoV-2 [84]. Slightly different to the above-mentioned assay, this test required denaturation and annealing of the RdRp oligo probe and the RNA sample. AuNPs were then added to the sample, which resulted in a colour change to blue if hybridization between the RdRp oligo probe and the target RNA sequence occurred. No colour change was observed when the RdRp oligo probe was added to the RNA samples from uninfected people or women infected with human papillomavirus. This was mainly attributed to the ssRNA adsorbing to the AuNPs, thus providing stability against salt-induced aggregation. The sensitivity (85.29%) and specificity (94.12%) of these assays was validated in clinical samples and compared to the RT-PCR test results. The assay had a LOD of 0.5 ng of SARS-CoV-2 RNA with a turnaround time of <30 min. Using the above-mentioned colorimetric assay, Alafeef et al. developed an RNA-extraction-free nano-amplified colorimetric test with a turnaround time of 40 min, which is visible with the naked eye and did not require prior sample processing, thus making the RNA extraction step optional. The newly improved diagnostic assay had an accuracy, sensitivity, and specificity of >98.4%, >96.6%, and 100%, respectively, with a LOD of 10 RNA copies/μL [102].

##### AuNP-Based LFAs

LFAs are immunochromatographic assays used for the detection and quantification of analytes in a complex mixture without the need for specialized and costly equipment. They have received considerable attention among researchers, both in the academic and industrial fields, because they are user-friendly, rapid, sensitive, cost-effective, and can easily be operated by non-specialized personnel [103], and, since the outbreak of the COVID-19 pandemic, there has been a renewed interest in these devices. Based on the analyte-antibody interaction, a distinct coloured line is formed on a test strip in the presence of the target [103]. Similarly, the LFAs have been used to detect the SARS-CoV-2 biomarker targets [85,86,104,105].

Using AuNP-based LFA, SARS-CoV-2 IgM antibodies were detected in COVID-19 human serum samples [85]. The IgM against the SARS-CoV-2 were captured by the anti-human IgM-AuNP conjugate at the conjugate pad, and the anti-human IgM-AuNPs/IgM complex flowed to the analytical membrane. The SARS-CoV-2 NP complex immobilized at the test line bound to the target IgM, while the goat anti-mouse IgG immobilized at the control line directly bound to the anti-human IgM-AuNPs. A positive test was indicated by the appearance of an intense red colour on both the control and test line within 15 min [85].

**Figure 3:**
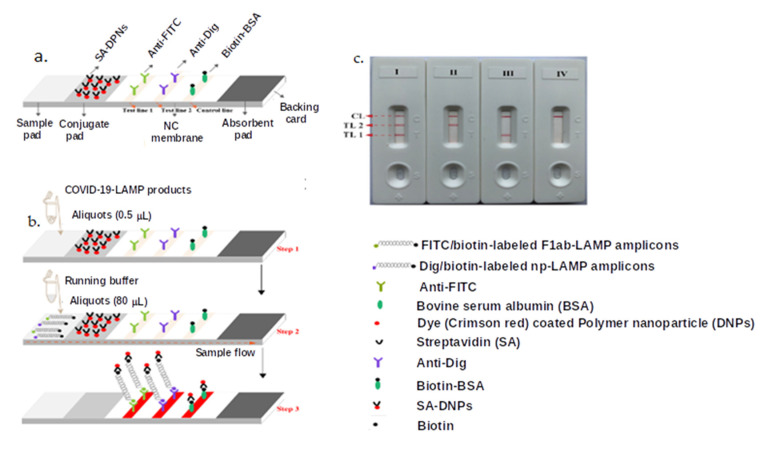
RT-LAMP-based LFA for detection of COVID-19. (**a**) The LFA design and assembly. (**b**) COVID-19 RT-LAMP products are added to the LFA. (**c**) The results are indicated as positive (I) when the ORF1ab and N (test line 1, test line 2, and control line appear on the LFB); (II) a positive result for N (test line 2 and control lines appear on the detection region); (III) a positive result for ORF1ab (test line 1 and control line appear on the detection region); or (IV) negative with only the control line visible [86].

LFAs can be incorporated with other techniques and synergistically increase their sensitivity and LOD. For example, a one-step RT-LAMP amplification coupled with SA-DNPs-based LFA (RT-LAMP-LFA) was developed for the detection of SARS-CoV-2 genes. LAMP primers for the ORF1ab and N genes of SARS-CoV-2 were simultaneously amplified in a single-tube reaction, and the results were quantified on a LFA test strip (Figure 3). The LOD of the SARS-CoV-2 RT-LAMP-LFA was 12 virus copies/25 µL reaction. The tests showed 100% sensitivity and specificity when used in COVID-19 clinical samples [86], whereas the commercially available LightMix Modular SARS-CoV RT-PCR had a 78.6% sensitivity [106].

#### 3.1.2. Fluorescence-Linked Immunoassay

Fluorescent molecules display a variety of measurable properties and have been incorporated in various immunoassays to increase detection sensitivity [107,108]. Generally, fluorophores conjugated to antibodies or antigens are excited by a laser and generate a fluorescent signal as an indicator of the presence of disease biomarkers. However, most conventional fluorophores have several limitations, such as low photostability, fluorescence quenching, and are affected by atmospheric ozone in the laboratory [109]. Quantum dots (QDs) have been widely utilized in place of conventional fluorophores owing to their broad excitation range, narrow emission spectra, high fluorescence quantum yield, large molar extinction coefficient, superior brightness, and photostability [92,110,111]. Taking advantage of the above-mentioned properties, a highly sensitive QD-linked immunoassay, utilizing QD nanobeads and magnetic iron oxide (MnFe_3_O_4_) nanospheres, was developed for the detection of human IgG in serum of COVID-19 patients. The MnFe_3_O_4_ coupled with mouse antihuman IgG was used as a capture probe, and the QD-conjugated rabbit antihuman IgG was used as a detection probe. This assay had a LOD of 4 pg/mL [87].

#### 3.1.3. NP-Based Biosensors

##### NP-Based Plasmonic Biosensor

**Figure 4:**
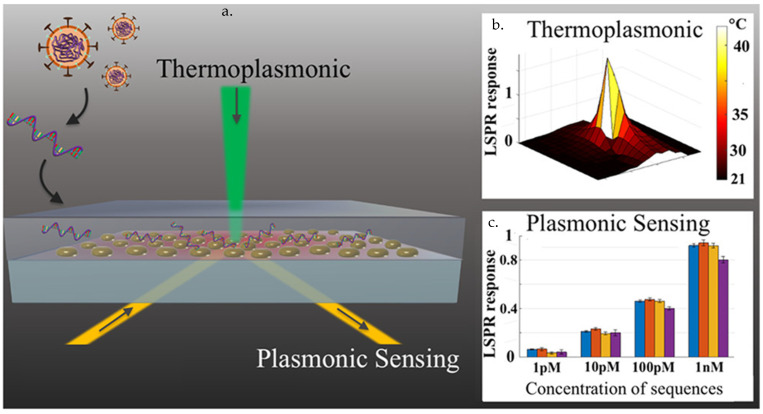
A dual plasmonic PT biosensor for sensitive detection of SARS-CoV-2 RNA sequence. (**a**) Schematic diagram of the dual-functional PT enhanced LSPR biosensing system. AuNIs were self-assembled on the glass surface and then functionalized with RdRp cDNA. (**b**) The localized PT heat is generated on the surface of the Au chip when irradiated at plasmonic resonance frequency, enhancing the hybridization temperature to facilitate binding of gene sequences. (**c**) The LSPR response to binding of viral sequences measured using the PT biosensor is concentration dependent [88].

Gold nanoislands (AuNIs) have been used for the development of plasmonic photothermal (PT) biosensors for detection of SARS-CoV-2 RNA sequences by targeting the E, RdRp, and the ORF1ab genes [88]. Due to their optical properties [112,113], MNPs produce heat when collective oscillations in the electron density at the surface of nanostructures arise via coupling to electromagnetic waves [114]. The amplified movement of the conduction electrons increases the frequency of collisions with the lattice atoms, resulting in the production of PT-induced heat. The above-mentioned dual-function plasmonic biosensor combines the AuNI’s PT effect and LSPR sensing transduction induced by hybridization of the cDNA complementary to the SARS-CoV-2 RNA target sequences (Figure 4). The thermoplasmonic heat (at 41 °C) on the AuNI chip induced in situ hybridization and discriminated between two similar gene sequences (RdRp genes from SARS-CoV versus SARS-CoV-2). The sensor exhibited high sensitivity, with a LOD of 0.22 pM [88].

A one-step label-free LSPR biosensor that had an extraordinary optical transmission effect for the rapid quantification of the SARS-CoV-2 pseudovirus was recently developed [115]. The biosensor was able to detect the SARS-CoV-2 pseudovirus in infected cells within 15 min and exhibited a LOD of 370 vp/mL. The pseudoviral particles were detected with low-cost handheld optical equipment controlled by a smartphone app. This demonstrated the feasibility of using the biosensor in PoC settings for rapid detection of SARS-CoV-2 infection [115].

##### Microfluidic-Based Sensors

The fundamental techniques for microfluidic device fabrication originated from the semiconductor industry, which was primarily based on silicon or glass materials [116]. With the advancement of technology over the years, this field has experienced massive progress and has developed several immunosensor platforms [117,118,119]. MNPs, especially AuNPs and AgNPs, have emerged as alternatives to the conventional silicon and glass used in these systems [120].

**Figure 5:**
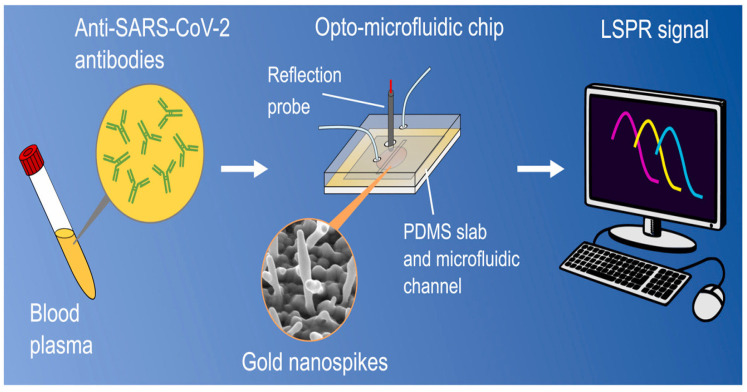
Gold nanospike-based opto-microfluidic sensor for the detection of anti-SARS-CoV-2. Human plasma containing the SARS-CoV-2 antibodies was collected in a tube. The blood plasma is laid on the opto-microfluidic chip that is electrodeposited with gold nanospikes. The shift in the LSPR of gold nanospikes is correlated with the amount of target antibodies [89].

In a NP-based microfluidic sensor, a change in interfacial refractive index, caused by the interaction between the antibody and an analyte, induces a shift in the LSPR that provides a detectable optical signal, as shown in Figure 5 [121,122]. Using this principle, an opto-microfluidic sensor for the detection of antibodies against the SARS-CoV-2 S protein was developed by electrodeposition of gold nanospikes onto a glass substrate and integrated in a microfluidic chip. The sensor was able to detect and quantify the amount of antibodies bound to the SARS-CoV-2 S protein within 30 min, with a LOD of 0.5 pM [89].

##### Field-Effect Transistor (FET) Biosensors

**Figure 6:**
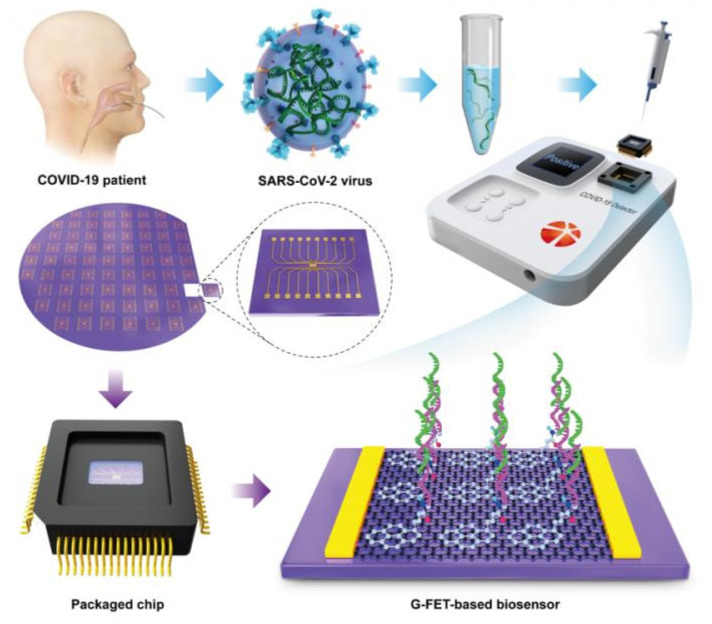
FET biosensor for diagnosis of COVID-19 at a PoC. Graphene was embedded on a sensing chip and SARS-CoV-2 S antibody was conjugated onto the graphene sheet. The sensing chip, which is connected to an electrical device, gives real-time response [126].

Nanomaterials have shown potential as transducers in the fabrication of FET biosensors. This is due to their excellent physical, optical, and electrochemical properties demonstrated in various biological applications [123,124]. The high mechanical strength, thermal stability, and outstanding conductivity of silicon- and carbon- (graphene and carbon nanotubes) based NPs are ideal for FET biosensors. Generally, the biorecognition molecules are immobilized on the surface of the NPs to capture disease-associated analytes. Binding of the analyte to the NP-conjugate generates an electrical potential that can be measured by a voltmeter [125]. The FET biosensors have the ability to detect the presence of an analyte at the PoC in a label free environment. The graphene-based FET biosensor, demonstrated in Figure 6, was used to measure antibodies against the SARS-CoV-2 S protein. The graphene oxide nanosheets increased the selectivity and sensitivity of the assay in various test samples, such as antigen protein, cultured virus, and nasopharyngeal swab specimens from COVID-19 patients. The biosensor detected SARS-CoV-2 in the culture medium and nasopharyngeal swab specimens, with a LOD of 16 pfu/mL and 242 copies/mL, respectively [90].

Using the same principle, Alafeef et al. [91] devised a rapid, cost-effective, and quantitative paper-based electrochemical biosensor coated with ssDNA-conjugated AuNPs for the detection of the SARS-CoV-2 virus. The biosensor was validated using Vero cells infected with SARS-CoV-2 and clinical samples. The sensor had a sensitivity of 231 copies/μL and a LOD of 6.9 copies/μL within 5 min [91]. To improve more on these biosensors, Yakoh et al. [127] developed a label-free paper-based electrochemical platform embedded with graphene-oxide for the detection of SARS-CoV-2 antibodies. The presence of SARS-CoV-2 antibodies interrupted the redox conversion of the redox indicator, resulting in a decreased current/voltage response. The biosensor exhibited a high sensitivity with a LOD of 1 ng/mL (three orders of magnitude more than the colorimetric LFA) within 30 min [127].

##### Optomagnetic Biosensor

Optomagnetic biosensors are homogeneous detection systems based on the optical and magnetic properties of MNPs in suspension. The ability to fine-tune the magnetic properties of MNPs makes them highly suitable to produce rapid high performance PoCT. Generally, MNPs functionalized with biorecognition molecules are mixed with multivalent target molecules and exposed to an external magnetic field. In the absence of the target molecules, the MNPs will assemble and disintegrate when the magnet is removed. In the presence of the target, the assembly is stable and remains intact even after the magnet is removed. Therefore, the optical properties of the MNPs in the presence and absence of the target molecule can be used to differentiate between the normal and diseased state [128].

**Figure 7:**
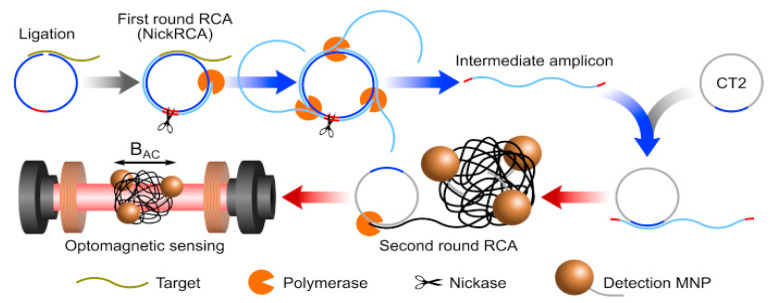
Homogeneous circle-to-circle amplification and optomagnetic biosensor. In the first round of rolling circle amplification (RCA), polymerases act tandemly to generate intermediate amplicons, which then anneal to the second round RCA, generating amplicon coils that lead to the assembly of MNPs. After a ligation step, all processes of amplification, hybridization, and detection take place simultaneously on an optomagnetic sensing-chip [92].

Using this principle, a biosensor based on the optomagnetic activity of IONPs was developed to quantify synthetic SARS-CoV-2 RdRp complementary DNA. In addition, the system integrated the homogeneous circle-to-circle isothermal nucleic acid amplification (Figure 7). In this assay, the ssDNA amplicons hybridize with the detection probes, which were grafted onto the IONPs, and this leads to a stable assembly of IONPs when subjected to a rotating magnetic field. The optomagnetic properties of untargeted-IONPs and the IONP-conjugates towards the scattering/absorption of light vary under the influence of an external magnetic field. The biosensor had a LOD of 0.4 fM and dynamic detection range of three orders of magnitude [92,129].

##### NP-Based Breathalyser Sensor Array

The ability to integrate multiple assays into one single device has set forth new dimensions of sensitivity, helping to estimate more parameters and improving the specificity of most sensors [130]. An example of a successfully integrated device is a sensor array, which consists of multi-analyte sensors, each of which converts energy into some electrical response, which is then interpreted using digital signal processing algorithms [131,132]. The NPs have unequivocally advanced developments in the sensor element design, allowing the development of real-time, sensitive, and portable systems that are able to target multiple analytes in a complex mixture [131].

**Figure 8:**
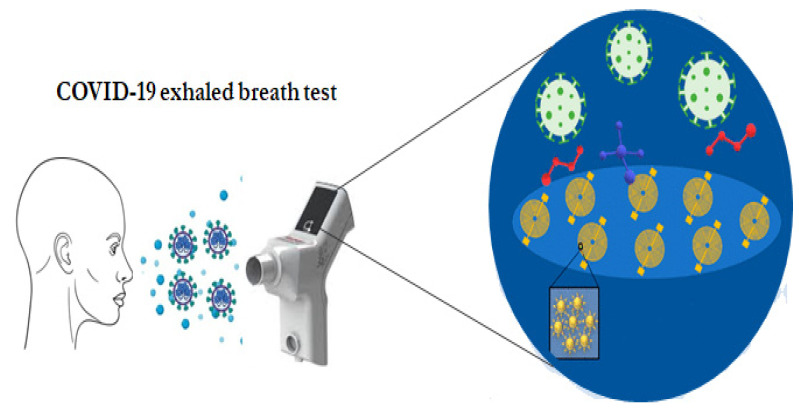
NP-based sensor array for the diagnosis of COVID-19. The sensor consists of different AuNPs linked to organic ligands, creating a diverse sensing layer that swells or shrinks upon exposure to VOCs, which lead to changes in the electric resistance [133].

A multiplex nano-based sensor array for the detection of COVID-19-specific biomarkers from exhaled breath was developed and used for rapid and accurate diagnosis of COVID-19 (Figure 8). The developed sensor was composed of different AuNPs linked to organic ligands, creating a diverse sensing layer. As the breath passes through the array, a mixture of COVID-19-related volatile organic compounds (VOCs) react with the sensors and emit a set of electrical resistance signals as a function of time. The sensor showed 94% versus 76% accuracy in differentiating COVID-19 patients from controls, and 90% versus 95% accuracy in differentiating between patients with COVID-19 and patients with other lung infections [133].

This type of assay presents a more convenient and non-invasive platform that can potentially increase compliance with COVID-19 testing. Sampling is much easier and does not require health professionals’ assistance as with the nasopharyngeal swabs, and it is very suitable for people of all ages, including children [134,135]. Studies have shown that, in addition to VOCs, the droplets collected from exhaled breath contain the virus as well. This concept was validated by using exhaled breath condensate (EBC) collected in RTube condensers or face masks to detect SARS-CoV-2 genes (S, E, N, ORF1ab) [135]. The EBC samples were compatible for testing with RT-PCR and aptamer-based electrochemical sensor [134].

## 4. Limitations and Challenges for Detection of SARS-CoV-2

Can nanotechnology entirely solve the challenges associated with SARS-CoV-2? Taking into account the infectious nature of this virus, the requirement for highly trained personnel to sample, handle, and dispose of patient test samples under strict biosafety guidelines will always be maintained. It will be a long while before these systems can be safely used and operated at a patient’s convenience. Like all the current systems, testing for molecular targets will require extraction of RNA and amplification of the genetic material in biosafety level two or three settings using specialized instruments. The pH and salt concentration in the RNA elution buffer might interfere with the nano-based assays and give false positive results when colorimetric systems are used [83,84].

Many of the presented systems, such as plasmonic PT, microfluidic, and FET biosensors, need external resources, such as optical and electrochemical sensing elements, and require expensive instruments and a high level of technical competence, thus making them unsuitable for use as PoCT. Furthermore, the signal generated from these techniques requires processing by advanced electronic devices or computing systems for accurate analysis of the test results [136].

Although the majority of the reported sensors have demonstrated excellent performance using viral cultures and particle isolates, the matrix effect may become more complex in the setting of an active SARS-CoV-2 infection. The presence of complex matrices found along the oral cavity may alter the final readout of the biosensor by either increasing the background or reducing the signal, consequently lowering the sensitivity of the assay [137]. The LFA and fluorescence-based immunoassay may also present challenges for the early diagnosis of COVID-19 due to the slow antibody response to SARS-CoV-2 viral infection. Hence, these tests are not suitable to detect individuals with mild or asymptomatic infections or those who are in the early stages of COVID-19 infection [138]. Some of the assays, such as RT-LAMP-LFA, are labour-intensive and time-consuming, making them unsuitable for PoCT.

This does not discredit the benefits of nanotechnology in any way as the nano-enabled diagnostic systems have shown high specificity and selectivity towards SARS-CoV-2. Moreover, the turnaround time required for these tests is shorter compared to the conventional tests. These nano-enabled diagnostic systems might be far from meeting the demands for PoCT, but nanotechnology brings hope for the development of rapid diagnostic tests that can shorten the turnaround time from 2 to 3 h of NAATs to 15 to 30 min for nano-enabled diagnostic systems. Although the nano-based optical, magnetic, fluorescence, and optomagnetic biosensors show potential for application in the diagnosis of COVID-19, the specialised equipment required for interpretation of the results often limits their use in PoCT. Nevertheless, colorimetric and breathalyser sensors have shown to be more user-friendly and do not require any complicated equipment nor assistance from medical personnel. Moreover, the results can be easily interpreted by the end-user based on the colour change or generated signal. Such systems make disease screening to be easy without putting a burden on the health care system. Additionally, they are cost-effective, selective, and able to detect SARS-CoV-2 at the early stages of infection when the viral load is still low.

## 5. Conclusions

The current and recurring global health and economic crisis associated with SARS-CoV-2 will continue to worsen unless there are improved testing systems and preventive measures in place. With the current diagnostic systems, the mild and asymptomatic cases, which account for 80% of the cases, are often misdiagnosed, suggesting that it could be more challenging to suppress the spread of this virus without more efficient diagnostic approaches. The scientific community has been racing to develop improved, sensitive, specific, and rapid SARS-CoV-2 detection devices that could be used for PoCT either at home or in resource-limited settings [139]. The early and rapid identification of infected patients can interrupt the chain of viral transmission by facilitating the isolation of infected individuals and allow quick provision of treatment options [140,141,142,143]. Studies have attempted to design various effective molecules against SARS-CoV-2 but the incidence of new variants and patient mortality will continue to increase without early and effective diagnosis [141,142,143].

The review provides an overview of recently developed nano-enabled diagnostic systems for COVID-19. Nanomaterials have been successfully incorporated in diagnostic devices for the diagnosis of COVID-19, such as colorimetric, PPT sensors, microfluidics-based sensors, FET sensors, fluorescent, and sensor arrays. Although they have shown a high specificity and selectivity against SARS-CoV-2, their implementation still lags behind and they fail to meet all the requirements of the WHO ASSURED (Affordable, Sensitive, Specific, User-friendly, Rapid and robust, Equipment-free and Deliverable to end-users) criteria. Nevertheless, these systems can help relieve the current pressure on RT-PCR-based diagnostic systems.

## Figures and Tables

**Table 1:** Clinical tests for diagnosis of SARS-CoV-2.

Parameters	NAATs *	Chest CT and Ultrasonography	Immunological Tests
Types	RT-PCR Nested RT-PCR Droplet digital PCR Loop-mediated isothermal amplification (LAMP)	X-ray	Enzyme-linked immunosorbent assay (ELISA) Chemiluminescence Enzyme Immunoassays (CLIA) Fluorescence Immunoassays (FIA) Lateral Flow Immunoassays (LFIA)
Analyte tested	Viral RNA sequence	lung scans	IgA, IgM, and IgG antibodies
Target	N, S, E, ORF1ab, RdRP, and nsp 14	SARS-CoV-2 features	M, N, and S proteins, and ORF1ab
Duration	2–6 h	15–120 s	15–30 min
Sensitivity	96%	97.2%	92% for IgA, 96% for IgG, and 98% for IgM

* Nucleic Acid Amplification Tests.

**Table 2:** Nanotechnology-based strategies for diagnosis of SARS-CoV-2.

Type	Nanoparticles	Target	Duration	LOD *	Ref.
Colorimetric assay	AuNPs	N	10 min	0.18 ng/µL	[83]
	AuNPs	RdRp	˂30 min	0.5 ng	[84]
Lateral flow assay (LFA)	AuNPs	IgM	15 min	-	[85]
	Dye streptavidin coated polymer nanoparticles (SA-DNPs)	ORF1ab and N	<2 min	12 copies/25 µL	[86]
Fluorescence-linked immunoassay	QDs and MnFe_3_O_4_ nanospheres	IgG	-	4 pg/mL	[87]
Plasmonic biosensor	Gold nanoislands (AuNIs)	E, RdRp and ORF1ab	-	0.22 pM	[88]
Microfluidic sensor	Au nanospikes	S	30 min	0.5 pM	[89]
Field-effect transistor biosensor	Graphene Oxide (GO) nanosheets	S	-	2.42 × 10^2^ copies/mL	[90]
	AuNPs	N	5 min	6.9 copies/μL	[91]
Optomagnetic biosensor	Iron oxide NPs (IONPs)	RdRp	100 min	0.4 fM	[92]

* LOD: limit of detection

## Data Availability

The review used information from published studies, which are referenced accordingly.
